# Pott's disease presenting with bilateral psoas abscesses in a resource-poor setting: Case report and literature review

**DOI:** 10.1016/j.ijscr.2025.111039

**Published:** 2025-02-10

**Authors:** Addisu Assfaw Ayen, Wondale Tsega Tebeje, Dagne Aschenaki Argaw, Tewodros Ayenew Yismaw, Gebeyaw Addis Bezie, Wali Ahmed Nur

**Affiliations:** a Department of Internal Medicine, Debre Tabor University, Debre Tabor, Ethiopia; b Department of General Surgery, Gerbo Primary Hospital, Somalia, Ethiopia; c Department of Internal Medicine, Bahir Dar University, Bahir Dar, Ethiopia; d Bachelor Degree Radiology Technology, Masters on public health, Chief Executive Officer, Gerbo primary hospital, Somalia, Ethiopia.

**Keywords:** Extra pulmonary TB, Psoas TB abscess, Pott's disease, Case report, Ethiopia

## Abstract

**Introduction and importance:**

Tuberculosis (TB) primarily affects the lungs, but can cause extrapulmonary TB (EPTB), including spinal TB (Pott's disease), which presents with variable symptoms. Tuberculous psoas abscesses, a complication of Pott's disease, are increasingly reported due to improved diagnostics.

**Case presentation:**

A 32-year-old male from the Somalia region of Ethiopia presented with a history of chronic lower back pain that had worsened over the preceding month, with lower back swelling, unquantified weight loss, and a slight limp. He also reported intermittent, mild lower quadrant abdominal pain. Physical examination revealed a chronically ill with stable vital sign. A 7 × 5 cm soft tissue swelling, localized to the lumbar area. Due to late presentation and the lack of advanced imaging capabilities such as CT scans at our facility, the diagnostic process was challenging. Spinal swelling was noted, and after incision and drainage, the discharge was analyzed using GeneXpert and *Mycobacterium tuberculosis* was detected by GeneXpert confirming a diagnosis of spinal tuberculosis, and an abdominal ultrasound showed a psoas abscess. The patient was started on anti-TB therapy and is improving,

**Case discussion:**

Tuberculous psoas abscess, a known complication of Pott's disease (spinal tuberculosis), is relatively uncommon, occurring in about 5 % of cases despite modern anti-TB treatment. Psoas abscesses can arise primarily or secondarily, the latter resulting from TB spread from nearby structures, as seen in our patient. Diagnosis of psoas abscess and spinal TB typically necessitates advanced imaging, which is currently unavailable in our setting. Timely management is crucial for improved patient outcomes. Management involves prolonged anti-TB therapy with pyridoxine supplementation and surgical intervention for neurological complications. Most patients respond well to this approach.

**Conclusion:**

Psoas TB abscess, while rare, poses a significant clinical challenge, particularly in resource-limited settings due to the patient's late presentation and the limited availability of advanced imaging, such as CT scans. Timely diagnosis, appropriate anti-tuberculosis therapy, and, when necessary, surgical interventions are crucial for optimizing patient outcomes.

## Highlights

• Pott's disease presenting with bilateral psoas abscesses;a rare presentation of Extra-pulmonary TB.
• Bilateral psoas abscesses; A rare complication of Pott's disease
• Challenges in resource-constrained environments in terms of diagnosis and management.
• Initiating appropriate anti-TB treatment early in the course of the disease is crucial for improving patient outcomes.

## Abbreviations

AFB: Acid-fast bacillus
TB: Tuberculosis
2RHZE: Isoniazid, Rifampin, Ethambutol, and Pyrazinamide for 2 months
10RH: Rifampicin and Isoniazid for 4 months
WBC: white blood cell
WHO: World Health organization

## 1 Introduction

Tuberculosis (TB) is an infectious disease primarily affecting the lungs, but it can also manifest in various organs outside the pulmonary system, a condition known as extrapulmonary tuberculosis (EPTB) [1]. Among the different forms of EPTB, spinal tuberculosis, or Pott's disease, involves the musculoskeletal system. The clinical presentation of Pott's disease is variable, ranging from mild back pain to severe neurological complications, such as lower extremity weakness [2]. While historically considered a relatively uncommon complication of Pott's disease, tuberculous psoas abscesses are increasingly being reported in recent literature. This apparent rise in incidence is largely attributed to advances in diagnostic capabilities, which have facilitated the identification of previously overlooked cases [3]. Late presentation, often stemming from factors such as low socioeconomic status, limited education, and inadequate healthcare infrastructure, poses significant challenges to diagnosis, treatment, and overall patient outcomes in resource limiting set ups. In this case report, we present the case of a 32-year-old male patient from the Somalia region of Ethiopia, who presented with a history of long-standing lower back pain associated with weight loss and bilateral psoas collections. The patient was subsequently diagnosed with Pott's disease complicated by bilateral psoas tuberculosis abscesses and treated with antitubercular therapy.

The case report narrated with Surgical Case Report (SCARE) 2023 guideline [4].

## 2 Case presentation

A 32-year-old male patient from the Somalia region of Ethiopia presented with a long-standing history of lower back pain. The pain had increased in intensity over the past month prior to presentation and was associated with mild swelling in the lower back, significant but unquantified weight loss, and a slight limp. He also reported mild, intermittent lower quadrant abdominal pain. The lower back swelling had progressively increased, becoming visibly noticeable. The patient denied any cough, fever, or generalized weakness. He also denied any known chronic medical conditions and reported no history of back trauma. On physical examination, the patient appeared chronically ill and emaciated. Vital signs were stable. Lymphadenopathy was absent. Chest auscultation revealed clear and resonant breath sounds. A 7 × 5 cm soft tissue swelling was noted in the lower back region, localized around the lumbar area and slightly to the right side. Tenderness was elicited upon palpation of the lower back region. The lower motor examination was normal, and other physical findings were unremarkable.

**Fig. 1:**
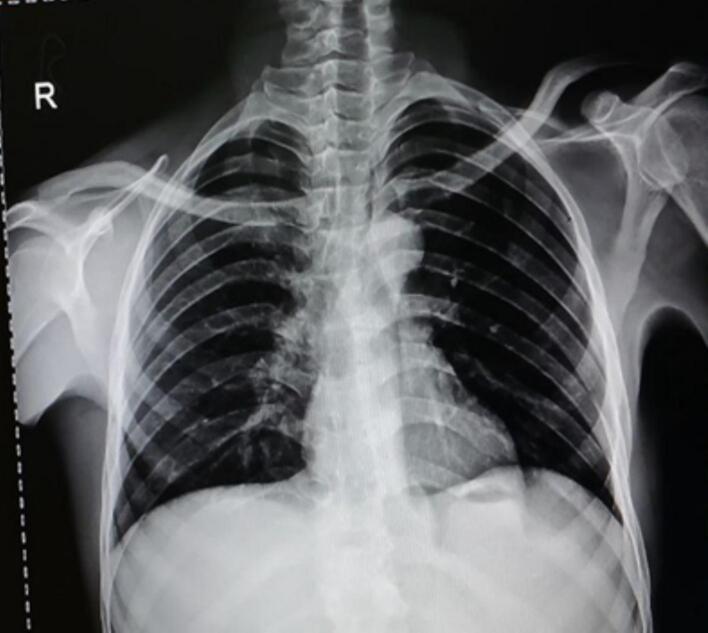
Normal Chest x ray.

**Fig. 2:**
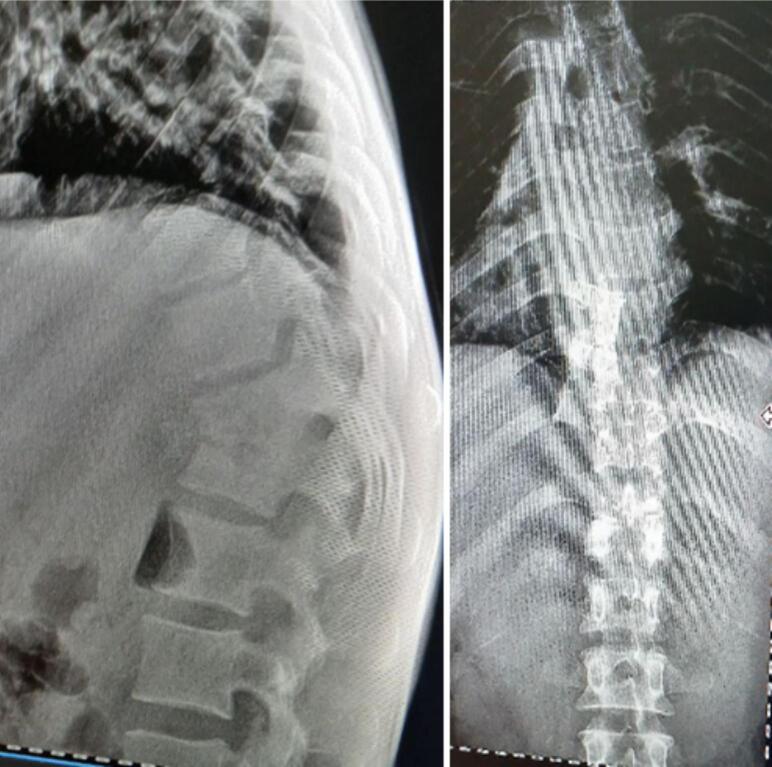
Thoracolumbar x ray showed Sclerosis, anterior wedging and hight loss of T11 and L1 vertebral bodies with a 3 cm measuring soft tissue attenuating perivertebral mass There is also blurring of lateral margin of the bilateral psoas shadow (psoas sign suggesting retroperitoneal mass).

**Fig. 3:**
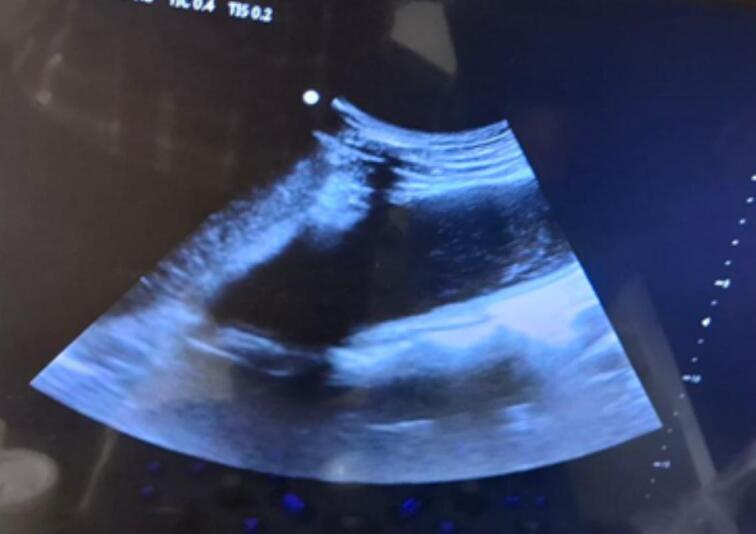
Abdomino-pelvic ultrasound showed 18 cm × 6 cm × 5 cm measuring echo complex left retroperitoneal collection on the left psoas muscle.

The patient underwent laboratory investigations, including a Complete Blood Count (CBC), which revealed mild anemia with a hemoglobin level of 11 g/dl and a low mean corpuscular volume (MCV) of 60 fL. The white blood cell (WBC) and platelet counts were within normal limits. The Erythrocyte Sedimentation Rate (ESR) was elevated at 40 mm/h. Baseline liver and renal function tests were within normal ranges. The patient tested negative for HIV. A chest X-ray was reported as normal (Fig. 1). A thoracolumbar X-ray is presented as Fig. 2, showed sclerosis, anterior wedging and hight loss of T11 and L1 vertebral bodies with a 3 cm measuring soft tissue attenuating perivertebral mass suggesting TB spondylodiscitis with prevertebral collection. There is also blurring of lateral margin of the bilateral psoas shadow (psoas sign suggesting retroperitoneal mass). An abdomino-pelvic ultrasound is presented as Fig. 3 showed 18cmx6cmx5cm measuring echo complex left retroperitoneal collection on the left psoas muscle. There was also a 5cmx3cm x 6 cm measuring echo complex Right psoas collection with a fistulous tract extending from the collection and opens to the skin at right lumbar area. An open surgical incision and drainage procedure was performed on the lower back collection, yielding approximately 1 l of serosanguinous fluid, which was sent for GeneXpert testing. *Mycobacterium tuberculosis* (MTB) was detected, with no rifampicin resistance identified while negative for gram stain and AFB.

Based on the clinical findings and investigations, a diagnosis of extrapulmonary tuberculosis (Pott's disease with bilateral psoas abscesses) was made. The patient was initiated on antituberculosis therapy with a regimen of 2 months of rifampicin, isoniazid, pyrazinamide, and ethambutol (2RHZE), followed by 10 months of rifampicin and isoniazid (10RH), in accordance with national and World Health Organization (WHO) guidelines. The patient is currently in his fourth month of antituberculosis treatment and is responding well, with a documented decrease in the size of the left psoas abscess. The right psoas abscess has resolved following drainage.

## 3 Discussion

Tuberculous psoas abscess, a known complication of Pott's disease, remains a relatively uncommon entity, reported to occur in approximately 5 % of cases, particularly in the era of modern antitubercular treatment [5]. However, significant challenges persist in resource-limited settings due to various factors, including delayed patient presentation and shortages in diagnostic capabilities [6]. Our patient's case exemplifies this, with a delayed presentation following a prolonged period of back pain, ultimately resulting in a psoas abscess as a late complication. TB, the second leading infectious cause of death globally after HIV/AIDS, presents with a wide range of clinical manifestations [7]. While skeletal involvement occurs in approximately 3 % of TB cases, a significant portion of these skeletal infections (around 50 %) are Pott's disease, also known as spinal TB [8].

Spinal tuberculosis can extend to adjacent soft tissues, leading to abscess formation through direct spread or lymphatic dissemination. Tuberculosis psoas abscesses can arise through two main pathways. Primary psoas abscess, a very rare form of TB, presents as an isolated abscess within the psoas muscle and may result from either direct infection or spread from a distant site via the bloodstream (hematogenous) or lymphatic system [9,10]. Secondary psoas abscesses, on the other hand, as our patient have result from the spread of TB from nearby structures, most frequently from the spine [9].

*Staphylococcus aureus* is the most common cause of psoas abscesses. While *Mycobacterium tuberculosis* is a less frequent cause overall, it is more commonly implicated in psoas abscesses in developing countries, though its prevalence is decreasing in the developed world [11]. Although our patient has no identifiable risk factors for a psoas abscess, this condition is more commonly seen in individuals with congenital or acquired immunocompromising conditions, such as HIV/AIDS, or those with chronic comorbidities [5]. Pott's disease can manifest with a spectrum of symptoms, ranging from mild to severe back pain that can lead to limping. Patients may also experience constitutional symptoms like fever and weight loss [6]. Our patient, for example, presented with lower back pain and a limp. In contrast, patients with psoas abscesses typically present with lower quadrant pain and hip pain, which can be exacerbated by active or passive hip extension [12].

Following a thorough clinical evaluation, the diagnosis of Pott's disease with psoas abscess relies heavily on both imaging findings and bacteriological confirmation of the specific causative organism through culture or PCR [3]. Our patient, for example, tested positive for *Mycobacterium tuberculosis* using GeneXpert following incision and drainage of the psoas abscess. However, due to logistical limitations, cultures were not performed. Given that a CT scan, the gold standard for diagnosing psoas abscesses [12], was unavailable in our setting, we utilized ultrasound to successfully identify and characterize the psoas collection, which correlated with our patient's findings. Vertebral MRI is the primary imaging modality for diagnosing Pott's disease; Plain vertebral X-rays can also be helpful in diagnosing Pott's disease, although they may be normal in the early stages. Later findings such as loss of disc height, vertebral body destruction, and endplate erosion are suggestive of Pott's disease [13,14].

Once a diagnosis of Pott's disease with a tuberculous psoas abscess is confirmed, management primarily involves prolonged anti-tuberculosis therapy, as recommended by the WHO, along with pyridoxine supplementation [15,16]. Patients who develop neurological complications may require surgical intervention [17]. Most patients, like ours, respond well to this management approach.

Untreated or late-presenting Pott's disease carries significant risks. Our patient's delayed presentation led to complications including a psoas abscess and others like; neurological deficits such as spinal cord compression, disseminated infection causing sepsis and arthritis, distant abscess formation, and even pelvic venous thrombosis (potentially due to compression or systemic inflammation). Early diagnosis and treatment are vital to prevent these complications through improved patient awareness and enhanced healthcare infrastructure [18].

## 4 Conclusion

Psoas abscess, a relatively uncommon complication of Pott's disease (spinal tuberculosis), can present with a wide range of clinical manifestations. Though infrequent in developed nations, this condition poses significant challenges in resource-limited environments due to factors such as limited diagnostic capabilities, delayed healthcare access, and restricted management approaches. Timely diagnosis and initiation of appropriate anti-tuberculosis therapy are critical for achieving good clinical outcomes. Patients experiencing neurological complications may require surgical intervention.

## Consent

Written informed consent was obtained from the patient for publication and any accompanying images. A copy of the written consent is available for review by the Editor-in-Chief of this journal on reques**t.**

## Ethical approval

Ethical approval for this study was provided by our institution ethical review committee.

## Research registration number

N/A.

## Declaration of Generative AI and AI-assisted technologies in the writing process

AI language modelling tools were utilized for the improvement of English-language only in this case report.

## Source of funding

There is no source of funding found for this paper.

## Declaration of competing interest

All authors declare that they have no conflict of interest.

## References

[1] Dass B.P.T.A., Watanakunakorn C. (2002). Tuberculosis of the spine (Pott’s disease) presenting as compression fractures. Spinal Cord.

[2] M. T. (2001). Spinal tuberculosis (Pott′s disease): its clinical presentation, surgical management, and outcome. A survey study on 694 patients. Neurosurgical Review..

[3] Abhishek Mewara KS, Dhillon,Meera Sharma. Molecular diagnosis of psoas abscess secondary to Pott's spine. Clinical Microbiology Newsletter. 2013;35(11):92-3.

[4] Sohrabi C.M.G., Maria N., Kerwan A., Franchi T., Agha R.A. (2023). The SCARE 2023 guideline: updating consensus Surgical CAse REport (SCARE) guidelines. Int J Surg Lond Engl..

[5] Chawla K.D.S.A., Mukhopadhayay C. (2012). Primary tubercular psoas abscess: a rare presentation. J Infect Dev Ctries..

[6] Mallick I.T.M., Rajendran T. (2004). Iliopsoas abscesses. Postgrad Med J..

[7] al. JLWe. (2010). Case report and review: Potts disease and epididymal tuberculosis presenting as back pain and scrotal mass. Am J Emerg Med..

[8] et.al. FCV. Spinal tuberculosis (Pott's disease) associated to psoas abscess: report of two cases and a literature review. Rev Soc Bras Med Trop. 2006;39(3):278-82. 10.1590/s0037-86822006000300011 16906254

[9] Shields D.R.P., Crowley T. (2012). Iliopsoas abscess–a review and update on the literature. Int J Surg..

[10] Finnerty R.U.V.J., Modarelli R.O., Buck A.S. (1981). Primary psoas abscess: case report and review of literature. J Urol..

[11] van den Berge SdM M., Kuipers T., Jansz A.R., Bravenboer B. (2005). Psoas abscess: report of a series and review of the literature. Neth J Med..

[12] Zissin R GG, Kots E, Werner M, Shapiro‐Feinberg M, Hertz M. Iliopsoas abscess: a report of 24 patients diagnosed by CT. Abdom Imaging. 2001;26(5):533‐9. 10.1007/s002610000201 11503095

[13] Cormican L.H.R., Messenger J., Milburn H.J. (2006). Current difficulties in the diagnosis and management of spinal tuberculosis. Postgrad Med J..

[14] DM F. Infectious spondylitis. Semin Ultrasound CT MRI. 2004;25:461-73. 10.1053/j.sult.2004.09.003 15663317

[15] Wong‐Taylor L‐A SA, Burgess H. Massive TB psoas absces. Case Reports. 2013;2013. 10.1136/bcr-2013-009966 PMC3670072 23696148

[16] A. B. Medical management of spinal tuberculosis: an experience from Pakistan. Spine (Phila Pa 1976). 2010;35:E787–E91. 10.1097/BRS.0b013e3181d58c3c 20581751

[17] Okada Y.M.H., Uno K., Sumi M. (2009). Clinical and radiological outcome of surgery for pyogenic and tuberculous spondylitis: comparisons of surgical techniques and disease types. J Neurosurg Spine..

[18] Maron R.L.D., Dobbs T.E., Geisler W.M. (2006). Two cases of pott disease associated with bilateral psoas abscesses: case report. Spine (Phila Pa)..

